# ﻿*Impatiensyingjingensis* (Balsaminaceae), a new species from Sichuan, China

**DOI:** 10.3897/phytokeys.242.119702

**Published:** 2024-06-06

**Authors:** Xinqiang Song, Boni Song, Mingxia Fu, Jiacai Wang, Jingyi Liu, Weirui Qin, Yuzhou Jiang, Leni Fan, Biao Yang

**Affiliations:** 1 Daxiangling Nature Reserve Management and Protection Center of Yingjing County, Ya’an Sichuan 625000, China Daxiangling Nature Reserve Management and Protection Center of Yingjing County Ya’an Sichuan China; 2 Key Laboratory of Bio-Resources and Eco-Environment of Ministry of Education, College of Life Sciences, Sichuan University, Chengdu, 610065, China Sichuan University Chengdu China; 3 The State-owned Forest Farm of Hongya County, Meishan 620360, China The State-owned Forest Farm of Hongya County Meishan China; 4 School of Life Sciences (School of Giant Panda), China West Normal University, Nanchong 637002, China Kent School Kent United States of America; 5 Kent School, Kent, CT06757, USА China West Normal University Chengdu China

**Keywords:** Balsaminaceae, Giant Panda National Park, *Impatiens*, new species, Yingjing County

## Abstract

This study describes *Impatiensyingjingensis* X.Q. Song, B.N. Song & Biao Yang, **sp. nov.**, a new species collected from the Yingjing area of the Giant Panda National Park. This new species is distributed at an altitude of 1400–2100 m, with a plant height of 30–130 cm. The flowers are purple-red or light purple red, with 3–9 flowers on each inflorescence and the dorsal auricle of the lateral united petals is thread-like and about 2 cm long, differing significantly from other species of *Impatiens*. Furthermore, molecular data, as well as micro-morphological evidence under SEM (of pollens), also support the establishment of the new species.

## ﻿Introduction

The genus *Impatiens* L. is the largest genus in the family Balsaminaceae, with more than 1,000 species (Grey-Wilson 1980; Yu et al. 2016; Song et al. 2021; Yuan et al. 2022). It predominantly inhabits the tropical and subtropical mountainous regions of the Eurasian continent and tropical Africa, with a minor presence in temperate zones of Eurasia and North America (Ren 2022). China is one of the five global hotspots for *Impatiens* diversity (tropical Africa, Madagascar, southern India and Sri Lanka, the eastern Himalayas and southeast Asia), accounting for more than 360 species (Chen et al. 2023). Most species are concentrated in southwest China, specifically in Yunnan, Sichuan, Guangxi, Guizhou and Xizang, showcasing narrow distributions and unique characteristics (Chen 2001; Yuan et al. 2022; Xiang et al. 2023).

In recent years, several new species and new records of this genus, including *I.longiaristata*, *I.tripetala*, *I.wawuensis*, *I.longlinensis*, *I.wutaishanensis*, *I.longshanensis*, *I.lihengiana* and *I.cavaleriei* have been discovered in southwest China (Ding et al. 2016; Xue et al. 2020; Liao et al. 2021; Peng et al. 2021; Song et al. 2021; Huang et al. 2023). These findings underscore the presence of numerous potential new species awaiting discovery within this diverse genus.

The Yingjing area of the Giant Panda National Park (GPNP) is located in Yingjing County, Sichuan Province, spanning a total area of 836 km^2^. It is situated in the mountainous area transitioning from the Sichuan Basin to the Qinghai-Tibet Plateau. The unique geographical position, varying altitudes and distinct climate conditions have given rise to a diverse array of flora and fauna, fostering complex ecological communities within the vicinity (Shao et al. 2022). During a survey in the Yingjing area of the GPNP from August to October of 2021, an intriguing *Impatiens* species was discovered thriving at the forest edge and in valleys at altitudes ranging from 1400 to 2100 m. It was similar to *I.lateristachys* in overall morphology, but after consulting a large number of specimens and investigating the morphological and micromorphological characteristics, significant differences were observed in flower, inflorescences, dorsal petal, lower petal, lateral united petals and lower sepal. Based on the combination of detailed field observation, morphological, micromorphological and molecular data, we confirmed that it represents a previously undescribed species. The primary objective of this research is to present a detailed description of this newly-discovered species.

## ﻿Materials and methods

### ﻿Plant sampling and morphological observation

Fresh plant material of the unidentified *Impatiens* species was collected from the Mount Yunwu area of Yingjing County, within the GPNP. The location was at Shaidianping (29°33.98'N, 102°45.00'E, 1624 m alt.). The collected specimens were deposited at the Herbarium of Sichuan University (Chengdu, China). Following the guidelines of Flora Reipublicae Popularis Sinica, Balsaminaceae, Tomus 47 (part 2) (Chen 2001) and Balsaminaceae of China (Yu 2012), various morphological characteristics of this species, such as plant height, leaf size and shape, inflorescence type, flower colour, pedicel length, petal width and stamen length, were meticulously observed and measured in the field. A comparative analysis with other *Impatiens* species was conducted. Additionally, colour photographs of the plants were taken and the inflorescences were dissected indoors. Furthermore, scanning electron microscopy was employed to observe the pollen of the plant.

### ﻿DNA extraction and sequencing

The fresh adult basal leaves of this species were collected in the field and then dried immediately with silica gel for the next step. Voucher specimens were stored at the Herbarium of Sichuan University (Chengdu, China) under deposition number 202108001. Firstly, we extracted the total genomic DNA from silica-dried leaves with a plant genomic DNA kit (Cwbio Biosciences, Beijing, China) referring to the manufacturer’s protocols. Then, the quality and quantity of extracted DNA were tested using 1% agarose gel electrophoresis and high-quality DNA was sequenced on Illumina NovaSeq platform at Personalbio (Shanghai, China) according to the standard Illumina sequencing protocols (Shendure and Ji 2008), with the sequence depth 6G. Paired-end 150 reads were obtained from libraries with an insert size of 300 bp. Finally, the software fastP v.0.15.0 (-n 10 and -q 15) (Chen et al. 2018) was used to filter the low-quality reads and gain high-quality reads.

In addition, total genomic DNA was also employed to amplify the Internal Transcribed Spacer (ITS) regions and the 30 µl amplification system was performed, which included 2 µl extracted total DNA, 10 µl ddH_2_O, 15 µl Taq MasterMix (CWBio, Beijing, China), 1.5 µl of 10 pmol µl^−1^ forward primers ITS4 (5’-TCC TCCGCT TAT TGATAT GC- 3’) and 1.5 µl of 10 pmol µl^−1^ reverse primers ITS5 (5’-GGA AGTAAA AGTCGT AAC AAG G-3’ (White et al. 1990). By executing the following programme: initial denaturation for 3 min at 94 °C, followed by 30 cycles of 45 s at 94 °C, 70 s at 54 °C and 90 s at 72 °C and then a final extension of 10 min at 72 °C, we obtained the amplified PCR products. Finally, we used a 1.5% (w/v) agarose TAE gel to examine all PCR products which were sent to Sangon (Shanghai, China) for sequencing. The software Geneious v.9.0.2 (Kearse et al. 2012) was used to edit the newly-sequenced ITS sequence and consensus sequences were gained.

### ﻿Plastome assembly and annotation

The plastome of this species was de novo assembled by NOVOPlasty v.2.6.2 (Dierckxsens et al. 2017) with the default parameters and *rbc*L sequence extracted from the plastome of *I.balsamina* (MZ902354) as the seed. The Plastid Genome Annotator (PGA) (Qu et al. 2019) was applied to annotate the plastome of this new species, setting the plastome of *I.balsamina* (MZ902354) as reference. Then, we manually corrected the start and stop codons and intron positions in Geneious v.9.0.2 (Kearse et al. 2012) based on the plastomes of congeneric species. The circular plastome map of this new species was drawn by the online Organellar Genome DRAW tool (OGDRAW) (Lohse et al. 2007). Finally, the newly-generated ITS sequence and plastome of this *Impatiens* species were submitted to the NCBI under accession numbers OR982404-OR982405 and OR978441, respectively.

### ﻿Phylogenetic analysis

To investigate the phylogenetic position of this species, 62 complete plastome data and 62 ITS sequences were employed to reconstruct the phylogenetic trees, and *Hydroceratriflora* was selected as outgroup (Suppl material 1). The plastome data and ITS sequences were straightway aligned with MAFFT v.7.221 (Katoh and Standley 2013) to generate the matrix, respectively. The matrixes were subjected to Maximum Likelihood (ML) and Bayesian Inference (BI) analyses. The ML analyses were inferred by adopting RAxML version 8.2.11 (Stamatakis 2014) with the GTRGAMMA model and 1000 replicates as suggested by the RAxML manual. The BI analyses were performed by using MrBayes v.3.2.7 (Ronquist et al. 2012) with the best-fit substitution model (GTR+I+G) for plastome data and (GTR+G) for ITS sequence determined by ModelTest v.3.7 (Posada and Crandall 1998) based on the Akaike Information Criterion (AIC). The Markov Chain Monte Carlo (MCMC) algorithm was run for 1,000,000 generations, sampling every 500 generations. The run finished when the average standard deviation of split frequencies was below 0.01. The first 25% of samples were discarded as burn-in and the remaining trees were maintained to yield the consensus tree. The phylogenetic trees of two analyses were visualised and edited by Interactive Tree of Life (iTOL) (Letunic and Bork 2019) and the nodes under 50% bootstrap support were collapsed.

## ﻿Results

### ﻿Morphological characteristics

**Figure 1.**
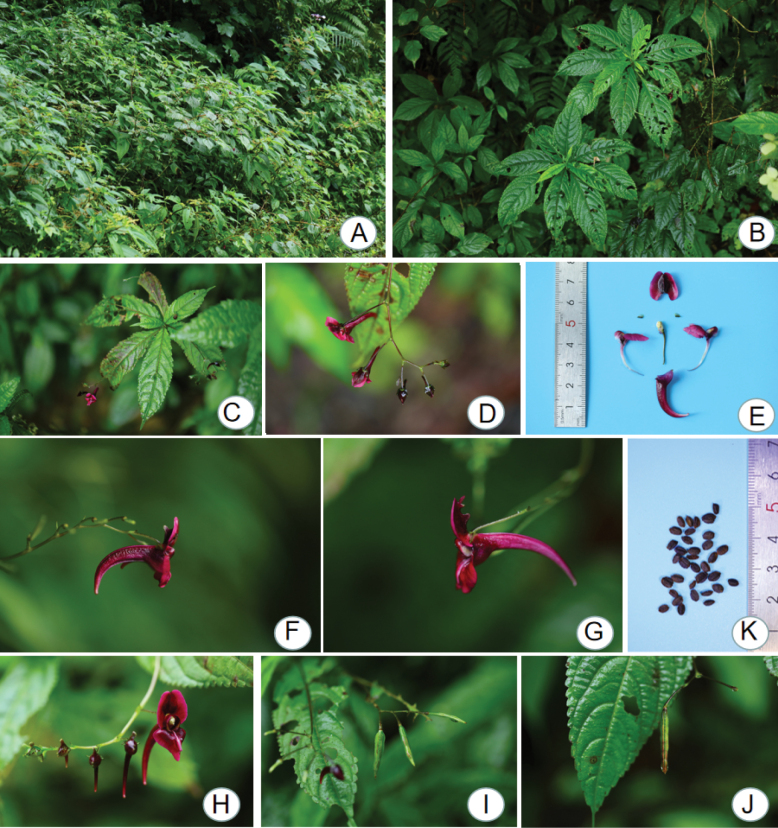
Habitat and morphology of *I.yingjingensis***A** habitat **B** plants **C** leaf **D** flower branch **E** the floral anatomy of *I.yingjingensis***F, G** flower, lateral view **H** flower, front view **I, J** capsule **K** seed.

We investigated carefully the morphology of this new species and observed that its distinctive morphological features are its flower, inflorescences, dorsal petal, lower petal, lateral united petals and lower sepal, such as it has purple-red or light purple-red flowers with 7–12 pairs of lateral veins. The inflorescences are axillary, slightly shorter than the leaf length or approximately equal to the leaf blade in length, 3–9 flowered arranged in a one-sided raceme on the inflorescence axis. The dorsal petal is orbicular approximately 15 mm in diameter, with a concave apex and obtuse tip, the mid-vein on the back thickening with narrow wings, wings 2-angled. The lower petal is gradually narrowing at the base into a sickle-shaped spur approximately 2 cm long; lateral united petals 2-lobed, auricle linear approximately 2 cm, elongate, inserted into spur (Fig. 1, Table 1).

**Table 1.** Comparative morphological characters of *I.yingjingensis* and related species.

Characters	*I.yingjingensis*	*I.siculifer*	*I.drepanophora*	*I.lateristachys*	*I.imbecilla*	*I.faberi*
Plant height (cm)	30–130	30–60	100	40–100	40–60	60–70
Leaf shape	ovate or elliptic	ovate-lanceolate or elliptic-lanceolate	ovate-lanceolate	obovate-lanceolate	ovate or ovate-oblong	ovate-lanceolate or elliptic
Leaf length (cm)	5–22	5–13	6–13	0.5–15	5–11	5–15
Leaf width (cm)	3.5–7	2.5–5	2–4	6	2.5–4	2.5–4.5
Length of petiole (cm)	0.5–4	1.5–3	5		2–4	2–4
Lateral veins	7–12	5–11	7–9	6–8		5–8
Inflorescence	unilateral cyme	cyme	cyme	unilateral cyme		
Pedicel length	12–29		15–20			10–20
Flower	3–9	5–8		3–6	2	2
Bracts	base, lanceolate	base, lanceolate	base, ovate-lanceolate	base, 2mm	ovate-lanceolate, 3–5mm	lanceolate, 2–3 mm
Flower colour	purple-red or light purplish-red	yellow	yellow	red, light red or white	light red	purple-red
Dorsal petal	orbicular, 15 mm, with a concave apex, the mid-vein on the back thickening with narrow wings, 2-angled	nearly circular, with the mid-rib on the back thickening into narrow wings	orange-yellow, slightly stalked	1.5–1.8 cm, top concave, with a blunt pointed head, deeply bifid at the base, the mid-vein on the back thickening with narrow wings, 2-angled	7–8 mm, with 2 shallow clefts at the top, the mid-rib on the back thickened, with a cockscomb-like projection	orbicular, 13–17 mm, concave or 2-cleft at the top, blunt, deeply bifid at the base, with thickened mid-rib on the back, with wings
Lateral united petals	2-lobed, auricle linear, 2 cm long, inserted into spur	2-lobed	2-lobed	auticula dorsalis in filum 1–1.5 mm latum, 1–1.3 cm long	2-lobed, auticula dorsalis in filum	2-lobed, auticula dorsalis in filum
Lower sepal	sickle-shaped, the eaves are boat-shaped, and the mouth is flat	narrowly funnel-shaped, with a beak-like short point at the apex	upper edge of the lip petal has a green elongated lobe	angular, 2.5–3 cm long, with an oblique blunt mouth	12–15 mm long; the mouth is oblique and tapering towards the tip, narrowing downwards	angular, 3–4cm, mouth is oblique, with a small point, bending inwards or straight from the middle
Spur	sickle-shaped spur, 2cm	introrse or extrorse stamens	long spirally inwardly curved	straight	straight or sickle-shaped	
Lateral sepals	ovate, 2–4 × 2 mm, with a pointed apex	narrowly elliptic, with an acute apex	sickle-shaped, 2 mm long, light green	diamond-shaped, about 2 mm long, with a truncated base and three veins	oval-shaped, 4 × 1.5–2 mm, with a long tapering tip at the top	green, egg-shaped, 6–8 × 3–5mm, with 3–5 veins and a thickened mid-rib
Flowering period	July to October	May to October	August		August to September	August to September
Capsule	clavate	clavate	clavate		clavate, 2.5–3 cm	Narrow linear , 2.5–3 cm

### ﻿Micromorphological observations of pollen

**Figure 2.**
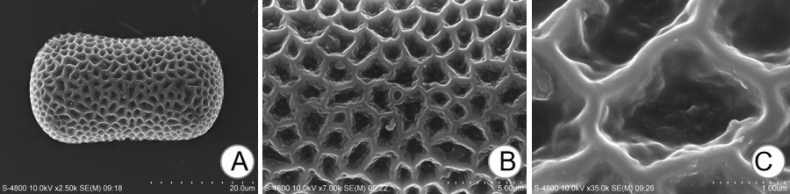
SEM images of pollen grains **A** polar view **B, C** partial view.

In further investigation of this species, we also observed its micromorphology of pollen grains under the scanning electron microscope. The results showed that the pollen grains of this species had a unique micromorphology, characterised by single-grain pollen with a flattened spherical shape. Its polar view was capsule-like, irregular and reticulated (Fig. 2).

### ﻿The structure and features of plastome

**Figure 3.**
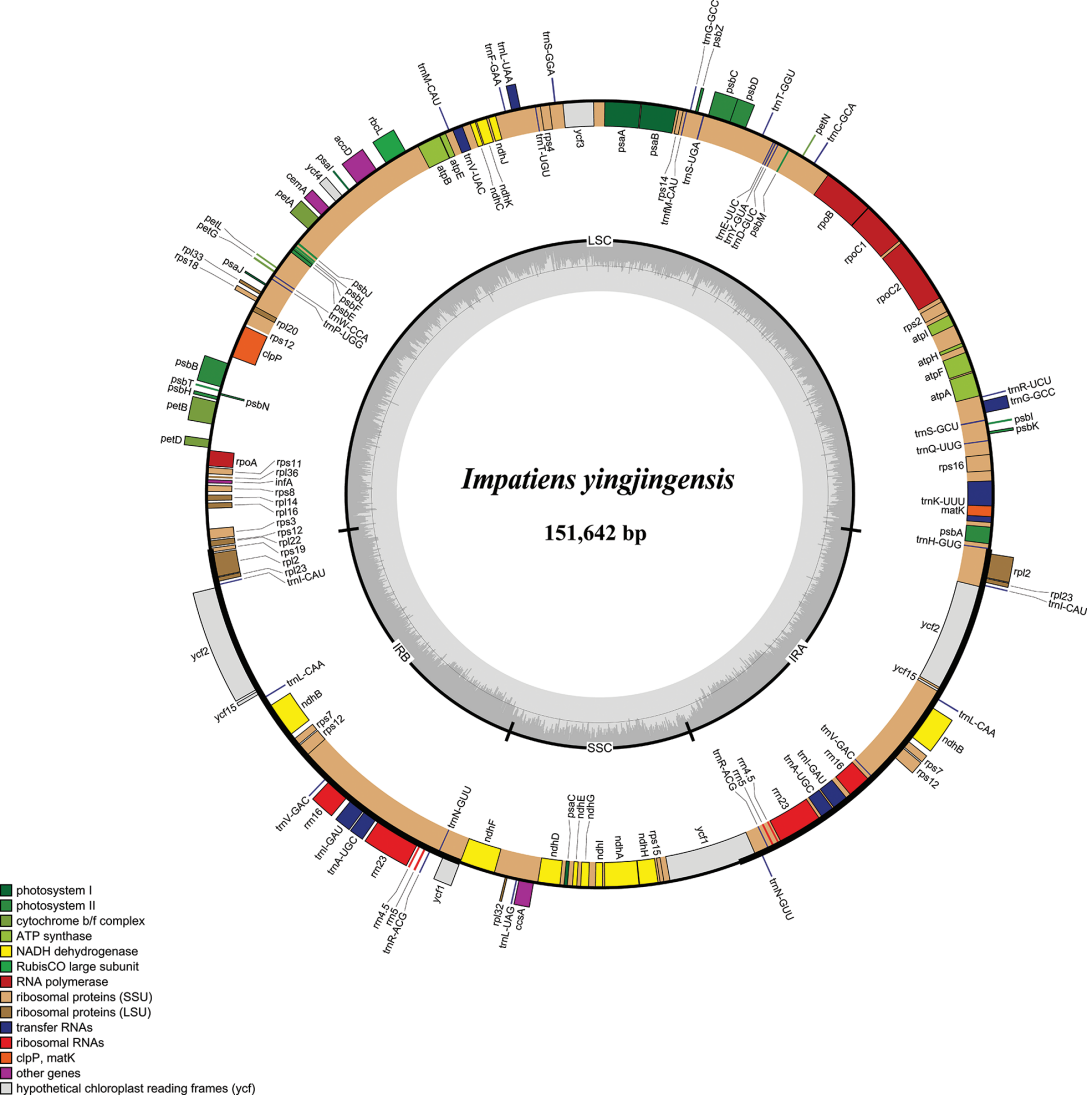
Plastome map of this new species. Genes shown in outside and insides of the circle are transcribed counter-clockwise and clockwise, respectively. The dark grey area of the inner circle denotes the GC content of plastome.

This species exhibited a typical quadripartite structure (Fig. 3), with a length of 151,642 bp, including a large single copy region (LSC: 82,588 bp), a small single copy region (SSC: 17,628 bp) and a pair of inverted repeat regions (IRs: 25,713 bp). The total GC content was 36.80%, with the GC content of the IRs regions being 43.1%, significantly higher than that of the LSC region (34.6%) and the SSC region (29.3%). The genome encoded 114 unique genes, including 80 protein-coding genes, 30 tRNA genes and four ribosomal RNA genes (Table 1). In addition, these unique genes had four categories: Self-replication, Genes for photosynthesis, other genes and Genes of unknown function (Table 2).

**Table 2.** Annotated unique genes information of *I.yingjingensis*.

Category of Genes	Group of gene	Name of gene
Self-replication	Ribosomal RNA genes	*rrn*4.5, *rrn*5, *rrn*16, *rrn*23
	Transfer RNA genes	*trn*C-GCA, *trn*D-GUC, *trn*E-UUC, *trn*F-GAA, *trn*G-GCC, *trn*G-UCC*, *trn*H-GUG, *trn*I-CAU, *trn*K-UUU*, *trn*L-CAA, *trn*L-UAA*, *trn*L-UAG, *trn*M-CAU, *trn*P-UGG, *trn*Q-UUG, *trn*R-UCU, *trn*S-GCU, *trn*S-GGA, *trn*S-UGA, *trn*T-UGU, *trn*T-GGU, *trn*V-GAC, *trn*V-UAC*, *trn*Y-GUA, *trn*W-CCA, *trnf*M-CAU, *trn*A-UGC*, *trn*I-GAU*, *trn*N-GUU, *trn*R-ACG
	Ribosomal protein (small subunit)	*rps*2, *rps*3, *rps*4, *rps*7, *rps*8, *rps*11, *rps*12**, *rps*14, *rps*15, *rps*16*, *rps*18, *rps*19
	Ribosomal protein (large subunit)	*rpl*2*, *rpl*14, *rpl*16*, *rpl*20, *rpl*22, *rpl*23, *rpl*32, *rpl*33, *rpl*36
	RNA polymerase	*rpo*A, *rpo*B, *rpo*C1*, *rpo*C2
	Translational initiation factor	*inf*A
Genes for photosynthesis	Subunits of photosystem I	*psa*A, *psa*B, *psa*C, *psa*I,*psa*J, *ycf*3**, *ycf*4
	Subunits of photosystem II	*psb*A, *psb*B, *psb*C, *psb*D, *psb*E, *psb*F, *psb*H, *psb*I, *psb*J, *psb*K, *psb*L, *psb*M, *psb*N, *psb*T, *psb*Z
	Subunits of cytochrome	*pet*A, *pet*B*, *pet*D*, *pet*G, *pet*L, *pet*N
	Subunits of ATP synthase	*atp*A, *atp*B, *atp*E, *atp*F*, *atp*H, *atp*I
	Large subunit of Rubisco	*rbc*L
	Subunits of NADH dehydrogenase	*ndh*A*, *ndh*B*, , *ndh*C, *ndh*D, *ndh*E, *ndh*F, *ndh*G, *ndh*H, *ndh*I, *ndh*J, *ndh*K
Other genes	Maturase	*mat*K
	Envelope membrane protein	*cem*A
	Subunit of acetyl-CoA	*acc*D
	Synthesis gene	*ccs*A
	ATP-dependent protease	*clp*P**
	Component of TIC complex	*ycf*1
Genes of unknown function	Conserved open reading frames	*ycf*2, *ycf*15

*: Gene with one intron. **: Gene with two introns.

### ﻿Phylogenetic analysis

**Figure 4.**
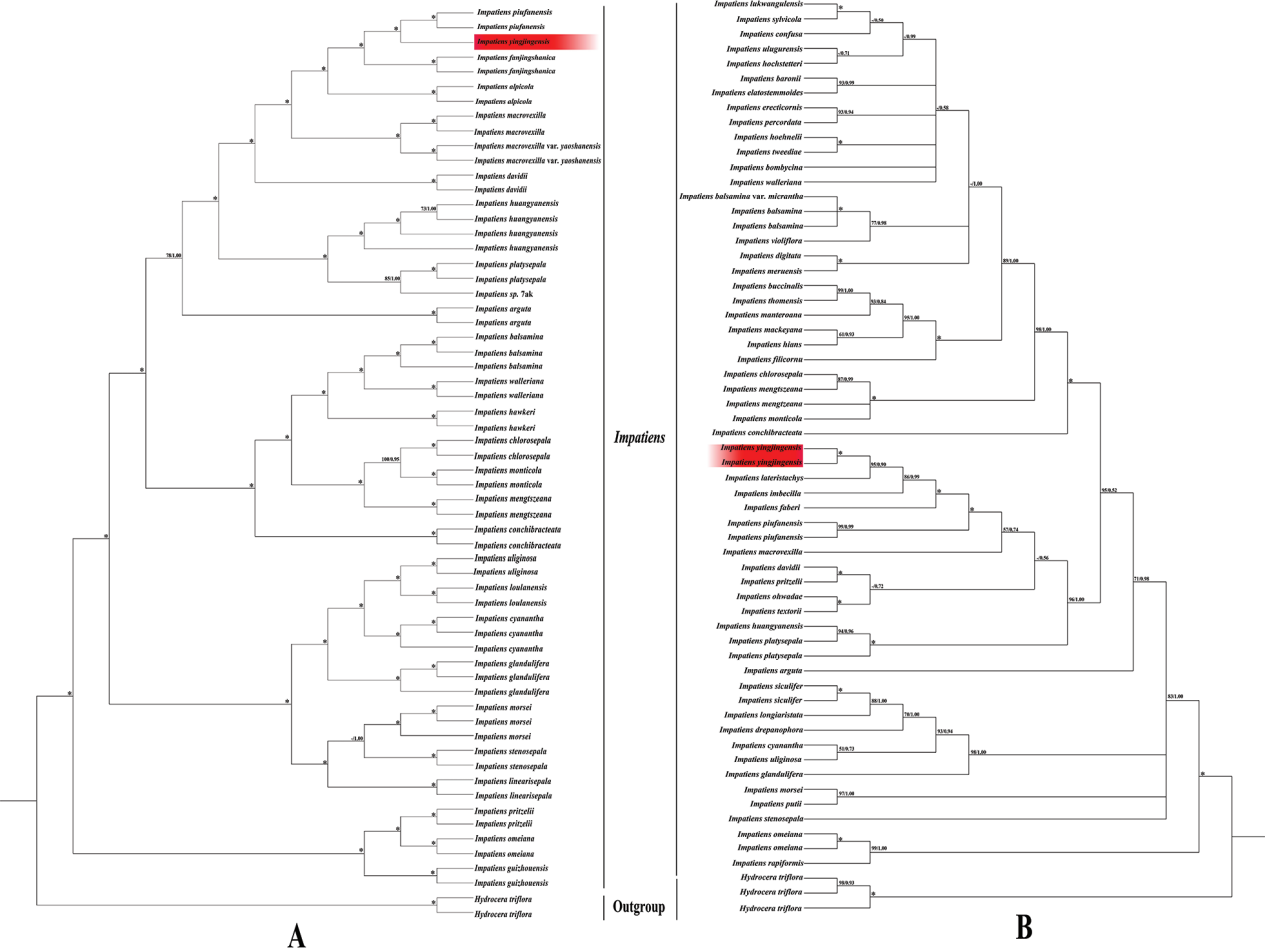
Phylogenetic trees constructed by Maximum Likelihood (ML) and Bayesian Inference (BI). The bootstrap values (BS) of ML and posterior probabilities (PP) of BI are listed at each node. (*) represents the node with PP = 1.00/BS = 100. “–“ means the values < 0.50/50. Red background indicates the newly-sequenced unknown *Impatiens* species **A** plastomes-based tree **B** ITS-based tree.

We employed 62 complete plastomes and 62 ITS sequences to reconstruct the phylogeny of this new species. Although the plastome data and ITS sequence yielded incongruent tree topologies, both strongly supported the fact that this new species clustered with other *Impatiens* members, belonging to the genus *Impatiens*. In the plastome-based and ITS-based phylogenetic tree, the results of the Maximum Likelihood (ML) and Bayesian Inference (BI) analyses generated well-resolved topologies and the topologies were highly identical as expected (Fig. 4). It was clearly observed that this new species formed a clade with *I.piufanensis* in the plastome-based tree (BS = 100, PP = 1.00) (Fig. 4A). However, in the ITS-based phylogenetic tree, this new species was clustered with *I.lateristachys* (BS = 100, PP = 1.00) (Fig. 4B). In addition, both phylogenetic trees also indicated that this new species was clearly distant from other related *Impatiens* members that were morphologically similar to it, including *I.faberi*, *I.drepanophora*, *I.siculifer* and *I.imbecilla* (Fig. 4).

## ﻿Discussion

China is recognized as a significant hotspot for the distribution of *Impatiens*. It offers a variety of habitat conditions for the genus *Impatiens* and breeds a variety of *Impatiens* resources, including regional endemics and Chinese-specific varieties. Due to the complexities associated with collection and identification, there is a lack of comprehensive and in-depth floristic surveys for the genus *Impatiens* in China. As a result, the resource status and phylogenetic relationships of the genus *Impatiens* in China, especially in key and vulnerable areas, are still unclear. Therefore, strengthening the floristic surveys and specimen collection of the *Impatiens* genus, especially in these critical and vulnerable regions, is an important task for the current research in taxonomy and floristics.

Through the investigation and research of the genus *Impatiens* in the Yingjing area, it has been found that the distribution points and population numbers of *I.yingjingensis* are relatively small, mainly being found in the valley, forest edge and roadsides at altitudes of 1400–2100 m in the areas of Mount Yunwu. Surveys and protection have not received adequate attention and it is essential to strengthen the investigation of the local resources of *I.yingjingensis* and conduct systematic research on the species diversity of the genus *Impatiens* in Yingjing County.

Through field observation and literature review, *I.yingjingensis* was found to bear the closest morphological resemblance to *I.lateristachys*, *I.drepanophora*, *I.siculifer*, *I.imbecilla* and *I.faberi*. However, their distinct differences were noted. The key features that distinguish *I.yingjingensis* and *I.lateristachys* are lower sepal and lateral sepals. Lower sepal in *I.yingjingensis* is sickle-shaped, the leaves are boat-shaped and the mouth is flat, while the lower sepal in *I.lateristachys* is angular with an oblique blunt mouth. The lateral sepals in *I.yingjingensis* are ovate with a pointed apex, while the ones in *I.lateristachys* are diamond-shaped with a truncated base and three veins, about 2 mm long. The most notable feature that distinguishes *I.yingjingensis* from *I.drepanophora* and *I.siculifer* is flower colour. *I.yingjingensis* has purple-red or light purplish-red flowers, whereas the flowers in *I.drepanophora* and *I.siculifer* are yellow. In addition, *I.yingjingensis* can be easily distinguished from *I.imbecilla* by the bracts. Bracts in *I.yingjingensis* are lanceolate, while *I.imbecilla* has ovate-lanceolate bracts. Additionally, the dorsal petal between *I.yingjingensis* and *I.imbecilla* is also different., The dorsal petal of *I.yingjingensis* is 2-angled, orbicular with a concave apex and the mid-vein on the back thickening with narrow wings, whereas two shallow clefts at the top, the mid-rib on the back thickened and a cockscomb-like projection of dorsal petal are observed in *I.imbecilla. I.yingjingensis* can be clearly distinguished from *I.faberi* by their lateral sepals. The ovate lateral sepals with a pointed apex are detected in *I.yingjingensis*, but green, egg-shaped and a thickened mid-rib of lateral sepals with 3–5 veins are found in *I.faberi* (Table 1). Furthermore, we also observed the pollen grains of *I.yingjingensis* and found they have unique micromorphology (single-grain pollen with a flattened spherical shape, polar view capsule-like, irregular and reticulated) (Fig. 2). Previous studies have illustrated that the pollen grains of different species of *Impatiens* show significant differences in size, equatorial view, polar view and exine thickness and these morphological characteristics serve as natural evidence for the systematic classification of the genus *Impatiens* (Zhang et al. 2014; Zeng et al. 2016; Zhang et al. 2023). Therefore, both the morphological and micromorphological features strongly support that *I.yingjingensis* is very different from other members of *Impatiens* and should be treated a new member of the genus *Impatiens*.

Although the explicit systematic position of *I.yingjingensis* remains undefined, both phylogenetic trees based on plastome data and ITS sequences, strongly supported that *I.yingjingensis* is nested within the genus *Impatiens*, indicating its affiliation with the genus. Consistent with Yu et al. (2016), *Impatiens* can be divided into I.subgen.Clavicarpa and I.subgen.Impatiens. The molecular phylogenetic analysis of *Impatiens* species, based on complete plastomes and ITS sequences, supported our proposed new species, to cluster into a clade which belongs to I.subgen.Impatiens. To confirm the phylogenetic position of *I.yingjingensis* within *Impatiens*, further molecular sequences, such as additional nuclear DNA fragments, are required in future studies. Thus, the molecular evidence further bolsters the argument that *I.yingjingensis* should be classified as a novel member of *Impatiens*. In conclusion, considering the evidence obtained from morphology, micromorphology and molecular evidence, the designation of *I.yingjingensis* as a new species of *Impatiens* is both logical and compelling.

### ﻿Taxonomic treatment

#### ﻿ Impatiens yingjingensis

Taxon classificationPlantaeEricalesBalsaminaceae

X.Q. Song, B.N. Song & Biao Yang sp.nov.

B4E8FA31-4806-520E-9277-950D985637C6

urn:lsid:ipni.org:names:77343115-1

##### Diagnosis.

*Impatiensyingjingensis* can be distinguished by the following morphological features from related species of *Impatiens*: purple-red or light purple-red flowers; inflorescence with 3–9 flowers; lower petal gradually narrowing at the base into a sickle-shaped spur approximately 2 cm long; lateral united petals 2-lobed, auricle linear approximately 2 cm, elongate and inserted into spur.

##### Type.

**Figure 5.**
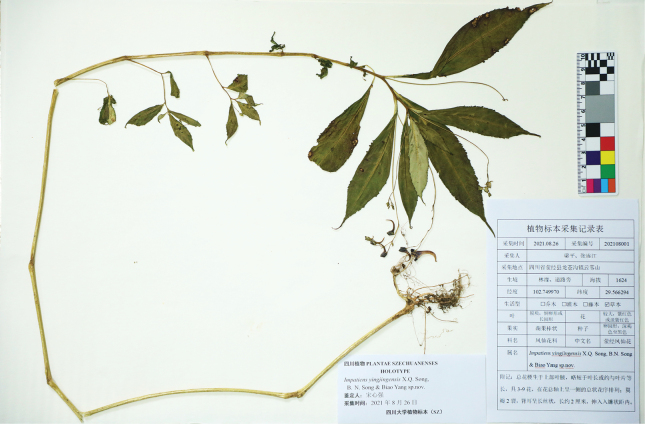
Holotype of *Impatiensyingjingensis* X.Q. Song, B.N. Song & Biao Yang, sp. nov.

China. Sichuan: Yingjing County, at the forest edge and in valleys, 29°33.98'N, 102°45.00'E, 1624 m alt., 26 August 2021, P. Liang & L.J. Zhang 202108001 (holotype: SZ;isotypes: SZ). (Fig. 5).

##### Etymology.

The species is named after Yingjing County, Sichuan Province, China, which is the type locality. The Chinese name is given as “荥经凤仙花”.

##### Description.

**Figure 6.**
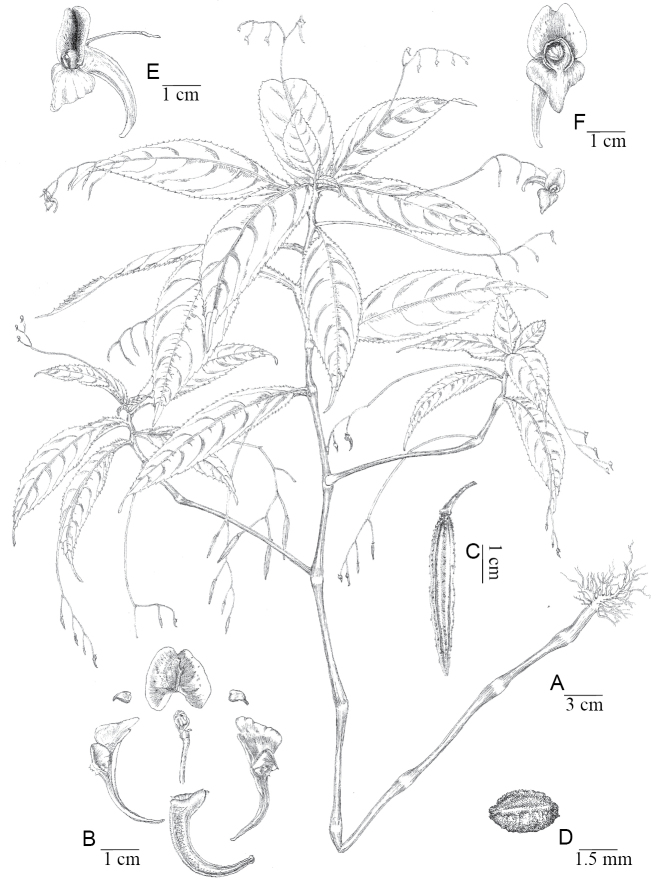
*Impatiensyingjingensis***A** plants **B** the floral anatomy of *Impatiensyingjingensis***C** capsule **D** seed **E** flower, lateral view **F** flower, front view. (Drawn by Liuqing Zhu)

Herbs annual, 30–130 cm tall, glabrous, stems fleshy, erect or ascendant, branched, basal nodes swollen adventitious roots. Leaves alternate, petiolate or subsessile on upper stem; leaf blades ovate-oblong, 5–22 × 3.5–7 cm, membranaceous, abaxially puberulent, with 2 stipitate glands at base, base cuneate, margin crenate, apex acuminate; lateral veins 7–12 pairs, petioles 0.5–4 cm long. Inflorescences axillary, slightly shorter than the leaf length or approximately equal to the leaf blade in length, unilateral cyme, 3–9 flowers; pedicels 12–29 mm long, with bracts above base; flowers relatively large, purple-red or light purple-red, 2–3.5 cm long; lateral sepals ovate, ca. 3 × 2 mm, entire, apex acuminate, mucronulate; dorsal petal orbicular, approximately 15 mm in diameter, with a concave apex and obtuse tip, the mid-vein on the back thickening with narrow wings, wings 2-angled; lower petal sickle-shaped, 2.5 cm long and the mouth is flat, gradually narrowing at the base into a sickle-shaped spur, approximately 2 cm long; lateral united petals 2-lobed, the lower lobe approximately 0.5 cm long, the upper lobe oblong and approximately 1 cm long, auricle linear and approximately 2 cm, elongate, inserted into spur. Capsule clavate, 2–5 cm long, hairless. Seed ellipsoid, dark brown to black (Figs 1, 6).

##### Phenology.

The flowering period is from July to October and the fruiting period is from August to November.

##### Distribution and habitat.

*I.yingjingensis* is distributed in Yingjing County, Sichuan Province, China, at altitudes of 1400–2100 m.

##### Additional specimens examined

**(paratypes).** China. Sichuan: Yingjing County, at the forest edge and in valleys, 29°36.76'N, 102°44.23'E, 1534 m alt., 24 August 2022, P. Liang & L.J. Zhang 202208001, P. Liang & L.J. Zhang 202208002, P. Liang & L.J. Zhang 202208003, P. Liang & L.J. Zhang 202208004, P. Liang & L.J. Zhang 202208005, P. Liang & L.J. Zhang 202208006 (SZ).

## Supplementary Material

XML Treatment for Impatiens yingjingensis

## ﻿Additional information

### Author ORCIDs

Xinqiang Song https://orcid.org/0009-0008-9818-2493

Biao Yang https://orcid.org/0000-0002-2181-8640

### Data availability

All of the data that support the findings of this study are available in the main text or Supplementary Information.

## Supplementary materials

Supplementary material 1 Plastomes data and ITS sequence included in phylogenetic analyses with GenBank accession Data type xlsx https://binary.pensoft.net/file/1063580This dataset is made available under the Open Database License (http://opendatacommons.org/licenses/odbl/1.0/). The Open Database License (ODbL) is a license agreement intended to allow users to freely share, modify, and use this Dataset while maintaining this same freedom for others, provided that the original source and author(s) are credited.Xinqiang Song, Boni Song, Mingxia Fu, Jiacai Wang, Jingyi Liu, Weirui Qin, Yuzhou Jiang, Leni Fan, Biao Yang

## References

[B1] Chen YL (2001) Balsaminaceae. Flora Reipublicae Popularis Sinicae Vol. 47(2). Science Press, Beijing, 1–243.

[B2] Chen S Zhou Y Chen Y Gu J (2018) fastp: An ultra-fast all-in-one FASTQ preprocessor. Bioinformatics (Oxford, England) 34(17): i884–i890. 10.1093/bioinformatics/bty560 PMC6129281 30423086

[B3] Chen Y Chen J Yong QQ Yuan TH Wang Q Li MJ Long SW Bai XX (2023) Species diversity and geographical distribution patterns of Balsaminaceae in China. Diversity 15(9): 1012. 10.3390/d15091012

[B4] Dierckxsens N Mardulyn P Smits G (2017) NOVO Plasty: De novo assembly of organelle genomes from whole genome data. Nucleic Acids Research 45(4): e18–e18. 10.1093/nar/gkw955 PMC5389512 28204566

[B5] Ding B Gadagkar SR Wang JC Zhang M Guo H Yu SX (2016) *Impatienswawuensis* (Balsaminaceae): A new species from Sichuan, China. Phytotaxa 273(4): 293–298. 10.11646/phytotaxa.273.4.5

[B6] Grey-Wilson C (1980) *Impatiens* of Africa. A. A. Balkema, Rotterdam.

[B7] Huang RX He BQ Chen Y Li MJ Bai XX (2023) *Impatienscavaleriei* (Balsaminaceae), a new species from the MiaolingMountains in Guizhou Province. Taiwania 68: 85–89. 10.6165/tai.2023.68.85

[B8] Katoh K Standley DM (2013) MAFFT multiple sequence alignment software version 7: Improvements in performance and usability. Molecular Biology and Evolution 30(4): 772–780. 10.1093/molbev/mst010 23329690 PMC3603318

[B9] Kearse M Moir R Wilson A Stones-Havas S Cheung M Sturrock S Buxton S Cooper A Markowitz S Duran C Thierer T Ashton B Meintjes P Drummond A (2012) Geneious Basic: An integrated and extendable desktop software platform for the organization and analysis of sequence data. Bioinformatics (Oxford, England) 28(12): 1647–1649. 10.1093/bioinformatics/bts199 22543367 PMC3371832

[B10] Letunic I Bork P (2019) Interactive Tree of Life (iTOL) v4: Recent updates and new developments. Nucleic Acids Research 47(W1): W256–W259. 10.1093/nar/gkz239 PMC6602468 30931475

[B11] Liao RL Cai L Yu ZY Wang YH Sun WB (2021) *Impatienswutaishanensis* (Balsaminaceae), a new species from Southeast Yunnan, China. PhytoKeys 176: 43–53. 10.3897/phytokeys.176.58825 33958938 PMC8065009

[B12] Lohse M Drechsel O Bock R (2007) Organellar Genome DRAW (OGDRAW): A tool for the easy generation of high-quality custom graphical maps of plastid and mitochondrial genomes. Current Genetics 52(5–6): 267–274. 10.1007/s00294-007-0161-y 17957369

[B13] Peng S Rono PC Yang JX Wang JJ Hu GW Wang QF (2021) Description of a new species and lectotypification of two names in Impatienssect.Racemosae (Balsaminaceae) from China. Plants 10(9): 1812. 10.3390/plants10091812 34579345 PMC8469104

[B14] Posada D Crandall KA (1998) MODELTEST: Testing the model of DNA substitution. Bioinformatics (Oxford, England) 14(9): 817–818. 10.1093/bioinformatics/14.9.817 9918953

[B15] Qu XJ Moore MJ Li DZ Yi TS (2019) PGA: A software package for rapid, accurate, and flexible batch annotation of plastomes. Plant Methods 15(1): 1–12. 10.1186/s13007-019-0435-7 31139240 PMC6528300

[B16] Ren LY (2022) Study on wild *Impatiens* resources and community species diversity in Southern Guizhou. Master’s thesis. Guizhou University, 2022.000853.

[B17] Ronquist F Teslenko M Van Der Mark P Ayres DL Darling A Höhna S Larget B Liu L Suchard MA Huelsenbeck JP (2012) MrBayes 3.2: Efficient Bayesian phylogenetic inference and model choice across a large model space. Systematic Biology 61(3): 539–542. 10.1093/sysbio/sys029 22357727 PMC3329765

[B18] Shao WJ Song XQ Chen C Zhao L Jin L Liao WB (2022) Diversity and distribution pattern of amphibians and reptiles in Yingjing Area of the Giant Panda National Park. Dongwuxue Zazhi 57(5): 707–721. 10.13859/j.cjz.202205007

[B19] Shendure J Ji H (2008) Next-generation DNA sequencing. Nature Biotechnology 26(10): 1135–1145. 10.1038/nbt1486 18846087

[B20] Song YX Xiao Y Peng S Cong YY Hu GW (2021) Two new species of *Impatiens* from China, and taxonomic insights into the Longifilamenta Group, which is endemic to China. Plants 10(8): 1697. 10.3390/plants10081697 34451742 PMC8398093

[B21] Stamatakis A (2014) RAxML version 8: A tool for phylogenetic analysis and post-analysis of large phylogenies. Bioinformatics (Oxford, England) 30(9): 1312–1313. 10.1093/bioinformatics/btu033 24451623 PMC3998144

[B22] White TJ Bruns T Lee S Taylor J (1990) Amplification and direct sequencing of fungal ribosomal RNA genes for phylogenetics. PCR protocols: a guide to methods and applications 18(1): 315–322. 10.1016/B978-0-12-372180-8.50042-1

[B23] Xiang NX Li ZF Li XY Wei CM Huang MJ Huang HQ (2023) Cloning and Expression Analysis of MYB61 Genes in *Impatienschlorosepala* and *Impatiensuliginosa*. Redai Zuowu Xuebao 44(8): 1561–1568.

[B24] Xue TT Xia CY Lidén M Xu WB Lu ZC Chen HL Li SW Yu SX (2020) Ignored biodiversity in acid soil islands in karst areas, south China: *Impatienslonglinensis* (Balsaminaceae), a new critically endangered species. Systematic Botany 45(4): 891–899. 10.1600/036364420X16033962925222

[B25] Yu SX (2012) Balsaminaceae of China. Peking University Press, Beijing.

[B26] Yu SX Janssens SB Zhu XY Lidén M Gao TG Wang W (2016) Phylogeny of *Impatiens* (Balsaminaceae): Integrating molecular and morphological evidence into a new classification. Cladistics 32(2): 179–197. 10.1111/cla.12119 34732016

[B27] Yuan TH Li MJ Ren LY Huang RX Chen Y Bai XX (2022) A dataset on the diversity and geographical distributions of wild *Impatiens* in China. Shengwu Duoyangxing 30(05): 118–122. 10.17520/biods.2022019

[B28] Zeng L Yan RY Zhang M Xu WB Zhang LJ Yu SX (2016) Taxonomic significance of the pollen morphology of sung. Clavicarpa (*Impatiens*, Balsaminaceae). Guangxi Zhi Wu 36(10): 1245–1252.

[B29] Zhang S Gao SP Zhang X He HF (2014) Pollen morphology and its relationship to taxonomy of 13 species in the *Impatiens* (Balsaminaceae) from Ya’an of Sichuan, China. Xibei Zhiwu Xuebao 34(3): 502–508. [In Chinese]

[B30] Zhang Q Zhao QY Gu ZJ Huang HQ Yan B Huang MJ (2023) Studies on pollen micromorphology of *Impatiens* plants in Southwest Sichuan. Yuan Yi Xue Bao 50(8): 1664–1678.

